# Inertia or unanticipated bottlenecks? Exploring stakeholder perspectives on the implementation determinants of the national alcohol policy five years post-enactment in Zambia

**DOI:** 10.1186/s13690-025-01737-7

**Published:** 2025-10-10

**Authors:** Adam Silumbwe, Miguel San Sebastian, Joseph Mumba Zulu, Charles Michelo, Klara Johansson

**Affiliations:** 1 Department of Health Policy and Management, School of Public Health, University of Zambia, PO Box 50110, Lusaka, Zambia; 2 Department of Epidemiology and Global Health, Umeå University, Umeå, 901 87 Sweden; 3 Department of Epidemiology and Biostatistics, School of Public Health, University of Zambia, PO Box 50110, Lusaka, Zambia; 4 Strategic Centre for Health Systems Metrics (SCHEME), Global Health Institute, Nkwazi Research University, PO Box 50650, Lusaka, Zambia

**Keywords:** Alcohol policy, Implementation, Determinants, Zambia

## Abstract

**Background:**

Alcohol use accounts for a huge proportion of the global burden of disease, and many countries grapple with its severe negative social and health consequences. In 2018, Zambia adopted a national alcohol policy with the aim to reduce the prevalence and impact of harmful alcohol use. However, five years post-adoption, the policy implementation has been slow. This study sought to explore the implementation determinants of the national alcohol policy five years post-enactment in Zambia. Implementation determinants, a concept from Bullocks theoretical framework, refer to the enabling and inhibiting factors that shape the alcohol policy and related implementation outcomes among implementing agents and teams.

**Methods:**

We employed a qualitative case study design using data collected from 25 semi-structured interviews targeting government ministries, civil society, and agencies responsible for enforcing the alcohol policy. We applied both inductive and deductive manifest thematic analysis using Bullock’s analytical framework for understanding the determinants of implementing evidence-based policies.

**Results:**

The alcohol policy was generally viewed by key stakeholders as comprehensive framework for action, yet its policy directives remained unclear. A restructured policy coordination committee enhanced the organisational capacity to implement joint action for this policy. However, weak collaboration between the council and state police hampered the enforcement of alcohol laws. Implementing agencies faced obstacles such as lack of financial and human resources, absence of rehabilitation services, and misapplication of alcohol selling licences. Further, community inaction and resistance to alcohol control laws affected the policy implementation. The socio-political environment contributed to implementation challenges through framing alcohol as an economic development issue and culturally tolerating harmful alcohol use. Additionally, changes in government affected policy ownership while unregulated illicit alcohol production and the sale of traditional and imported spirits further hampered the implementation of the policy.

**Conclusion:**

Overcoming implementation hurdles to the alcohol policy demands comprehensive strategies such as engaging communities, challenging cultural norms, strategically assigning funding, and fostering collaboration among implementing agencies. This may entail leveraging enablers such as stakeholder recognition of the policy framework and the restructured committee for the coordination of the alcohol policy implementation. Key actions should include empowering local government to enforce measures to reduce unregulated alcohol availability and ensure adequate resource allocation for alcohol control activities among implementation agents, including those providing treatment and rehabilitation services.

**Supplementary Information:**

The online version contains supplementary material available at 10.1186/s13690-025-01737-7.

**Table:** 

**Text box 1. Contributions to the literature**
• This study unpacks several contextual factors that have shaped implementation of the alcohol policy in Zambia five years post-enactment using an analytical framework that adopts a multilevel view of policy implementation determinants by Bullock et al.
• The implementation determinants affecting the alcohol policy ranged from the ambiguity in the policy content, siloed interorganisational relationships, poor response from those affected by the policy to a socio-political context that is unsupportive of the alcohol policy implementation.
• Understanding these implementation determinants is crucial for developing tailored implementation strategies to overcome bottlenecks at different levels of governance and realign efforts with the alcohol policy’s vision.

## Background

Annually, harmful alcohol use accounts for approximately three million deaths, contributing to 5.1% of the global burden of disease [1]. Among those aged between 15 and 49 years, alcohol is the foremost driver of premature mortality and disability [2]. In Africa, alcohol is responsible for 12.5 million disability adjusted life years lost among men according to data from 2012 [3]. The African region has a large proportion of non-drinkers, primarily driven by religious influence, especially countries in the West and North of Africa that have larger Muslim populations for whom alcohol consumption is prohibited by religion. However, among drinkers it boasts the highest per capita consumption, with 32.2% of the population being current drinkers [1]. About 19% of sub-Saharan African (SSA) men are binge drinkers – they consume large volumes of alcohol on a single occasion [1]. SSA is projected to experience a surge in harmful alcohol use driven by rapid urbanization, economic growth, increased alcohol availability, and lax alcohol policies [4].

The 2010 Global Strategy to Reduce Harmful Alcohol Use by the World Health Organization (WHO) promotes adoption of cost-effective interventions – ‶Best Buys [5].″ These interventions include higher taxes, restricted availability and marketing of alcohol, enforcement of drinking and driving laws, and the treatment of alcohol use disorders. Despite the adoption of the WHO strategy and the subsequent WHO Global Action Plan for the Prevention and Control of Non-Communicable Diseases in 2013, the Africa region has witnessed limited progress in the implementation of these interventions [6, 7]. The majority of African countries maintain low excise taxes and have not comprehensively adopted all of the ‶Best Buys [7, 8].″ In a few cases where countries have adopted the Best Buys, implementation has been very weak [4, 9]. Cultural beliefs that favour excessive drinking, insufficient funding, industry influence, poor coordination among oversight institutions, and social stigma hamper implementation of alcohol policies in SSA [8, 10].

Like other SSA countries, Zambia grapples with the severe negative social and health consequences of harmful alcohol use [11, 12]. Alcohol is the biggest single cause of road traffic fatalities, as well as the main driver of intimate partner sexual violence and psychiatric admissions at the country’s largest and only mental hospital – Chainama Hills Mental Hospital [13, 14]. According to the 2016 WHO report, the prevalence of alcohol use disorders in Zambia was about 5.5%, which surpassed the average of 3.7% for the WHO African Region [15]. Further, our recent nationally representative study found the prevalence of binge drinking to be approximately 12%, and significantly higher in men than women in Zambia (19% vs. 5%) [16].

In 2018, the Zambian government adopted an alcohol policy premised on the WHO strategy and action plan, with the ambitious goal of substantially reducing the prevalence and impact of harmful alcohol consumption by 2030 [17]. Central to this policy was the establishment of a National Alcohol Policy Coordination Committee, tasked with engaging multiple sectors in the comprehensive implementation, monitoring, and evaluation of alcohol control activities. The policy aims to strengthen and enforce existing legislation, such as the Liquor Licensing Act No. 20 of 2011, which regulates the production, distribution, and retail sale of alcohol, as well as the Road Traffic Act of 2022, specifically targeting drunk driving (driving with blood alcohol concentration above 0.38 mg/mL). Additionally, the policy mandates that all alcohol sold in Zambia must be distributed through licensed outlets and adhere to stringent safety and quality standards. It also emphasizes the importance of providing services for the treatment of alcohol use disorders and associated harms, while actively working to reduce the stigma faced by individuals undergoing treatment and rehabilitation. Lastly, the policy advocates for integrating culturally acceptable traditional values into all alcohol control efforts, fostering a culturally rooted response in the fight against harmful alcohol use. All these policy measures were operationalized in the alcohol policy implementation plan that provides a framework of activities to all stakeholders working in alcohol control.

While the adoption of the alcohol policy was widely lauded by both the Zambian government and the civil society organizations that supported the process, the initial enthusiasm has not materialized into visible gains in control of harmful alcohol use five years later. For example, even with the adoption of the policy, enforcement of existing alcohol regulations—covering production, distribution, advertising, sponsorship, and sales promotion—has largely remained suboptimal, significantly hampering the policy’s effectiveness [15]. As with other SSA countries, the alcohol policy has encountered several huddles specific to the Zambian context. However, there is still a notable scarcity of studies that investigate factors influencing the alcohol policy implementation. Understanding these factors is crucial for developing tailored implementation strategies to overcome bottlenecks and realign efforts with the policy’s vision. Ultimately, these insights will contribute to enhance future implementation efforts of this policy in Zambia.

Therefore, this study sought to explore the implementation determinants of the national alcohol policy five years post-enactment in Zambia. To do so, we applied Bullock’s analytical framework for unpacking the determinants of implementing evidence-informed policies and practices [18]. In this context, the determinants consist of specific enabling and inhibiting factors that shaped the alcohol policy and related implementation outcomes among implementing agents and teams [19].

### Study framework

**Fig. 1:**
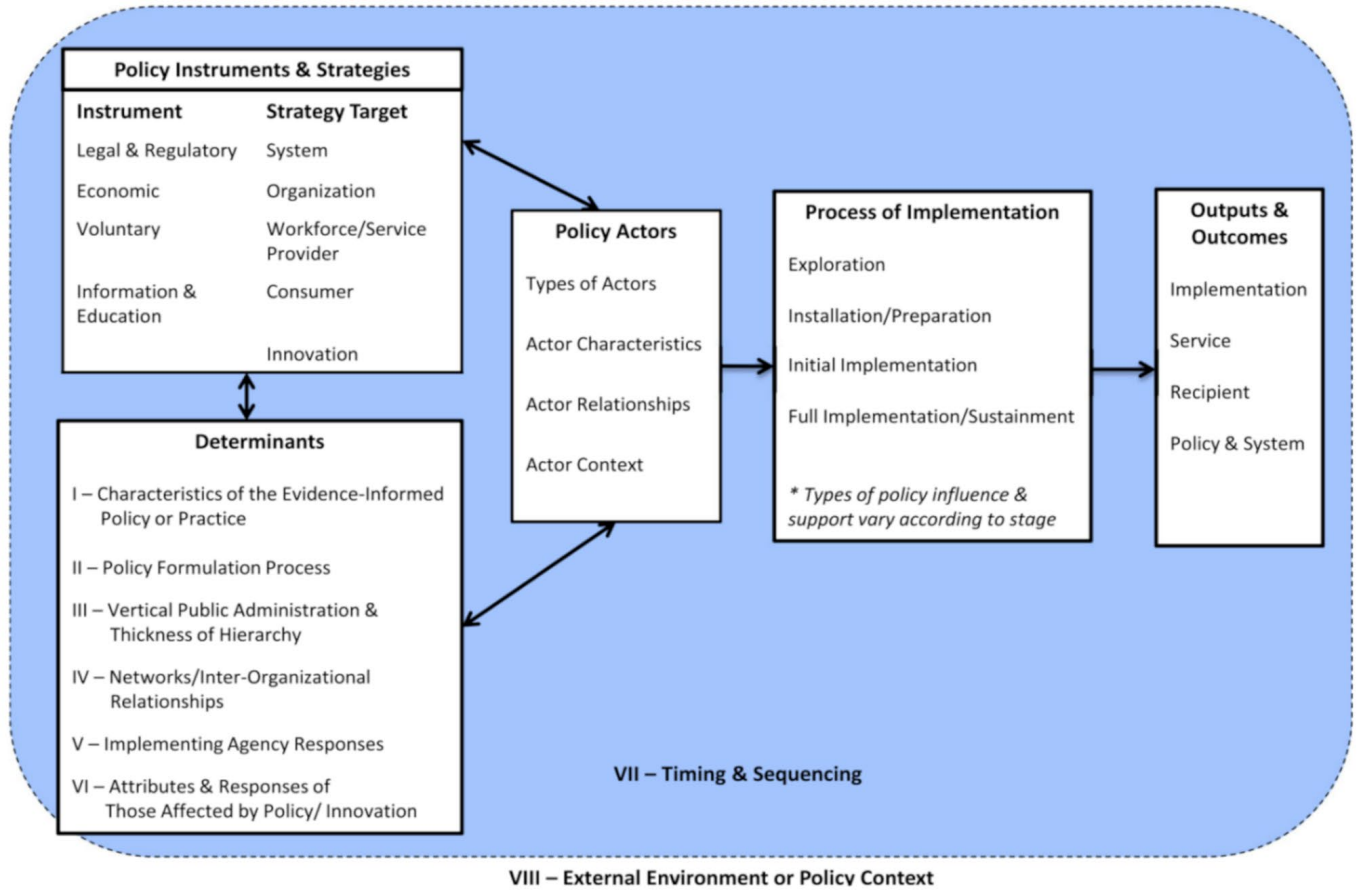
Policy implementation determinants framework [15]

Bullock’s framework identifies three key sets of policy-related factors that influence the implementation process: policy instruments and strategies, determinants of implementation, and policy actors (Fig. 1) [18]. This paper specifically focuses on examining the determinants of implementation, starting from when the National Alcohol Policy was adopted. The framework outlines six categories of determinants across different levels, including the characteristics of evidence-informed policy, network and interorganizational relationships, responses from implementing agencies, attributes of those affected by the policy, and the broader external environment or policy context (see Determinants box: Fig. 1). By adopting a multilevel view of the implementation determinants, this framework allowed us to unpack several contextual factors shaping implementation of the alcohol policy in Zambia.

## Methods

### Study context – alcohol policy development and implementation

The government through the Ministry of Health (MoH) coordinated and superintended over the alcohol policy development process and was envisioned to be the custodian of this policy. The CSOs through the Zambia Network Against Harmful Use of Alcohol, comprising of international partners, anti-alcohol and faith-based organizations, played a crucial role by providing financial and technical resources [17].

The adoption of the alcohol policy was followed by the establishment of a national alcohol policy coordination committee chaired by the MoH, and the Ministry of Local Government and Rural Development (MLGRD) serving as the secretariat. Government Ministries such as Commerce, Trade and Industry, Transport and Communication, Finance, Justice as well as government agencies such as the Zambia Bureau of Standards were also co-opted. Initially, this committee had almost 40 members. However, they had not met regularly since 2018 due to the failure to form a quorum. As of 2023, when we collected data, the committee had been reorganised into five core government ministries and a few CSOs.

### Study design

We employed a qualitative case study design, which according to Yin’s classification of case studies is an approach that explores and describes a specific phenomenon in detail. Since this analysis is applied to a particular policy in a specific geographical area, a qualitative case study design is most appropriate. In this study, the case consisted of the alcohol policy, its implementation activities, and the associated context [20]. We were guided by the consolidated criteria for reporting qualitative research (COREQ) guidelines (additional file).

### Study setting

We conducted the study in Lusaka, the capital city of Zambia, primarily due to the concentration of key policy agencies and stakeholders that are integral to implementation of the alcohol policy. The CSOs involved in this study are engaged in prevention and treatment of harmful alcohol use and related disorders. Government institutions varied, ranging from those solely responsible for policy oversight to those implementing specific laws aimed at mitigating the impact of harmful alcohol use. The latter category included the Road Transport and Safety Agency, the Drug Enforcement Commission, and the country’s sole mental health referral hospital, which treats people with alcohol use disorders (Table 1).

**Table 1:** Study participants organizations

Participant Category	Role in Policy Implementation	Number of Interviews
Civil Society • Alcohol Concern Zambia • Anti-alcohol Zambia • Pioneer Abstinence Association of the Sacred Heart of Jesus • Serenity Harm Reduction Programme Zambia • People Affected by Alcohol Abuse • Anti-Alcohol and Drug Abuse Zambia • Paradigms Zambia	Policymakers and implementors	7
Chainama Hills College Hospital (mental health hospital)	Treatment and rehabilitation of alcohol use disorders	3
Lusaka City Council	Enforcement of Liquor licensing Act of 2011	3
Road Transport and Safety Agency	Enforcement of the Road Traffic Act of 2022	3
Drug Enforcement Commission	Treatment and rehabilitation of alcohol use disorders	1
Ministry of Health	Policymaker and implementors	6
Ministry of Local Government and Rural Development	Policymaker and implementors	1
Ministry of Education	Policymakers and implementors	1
**Total interviews done**		**25**

### Participant selection and recruitment

We employed purposive and snowball sampling. Firstly, we enlisted stakeholders in the alcohol policy implementation coordination committee comprising representatives of the MLGRD, MoH, Ministry of Education, Ministry of Trade and Commerce, and some CSOs. Secondly, we used snowballing [21], an approach where the selected stakeholders referred us to recruit other participants responsible for the enforcement of specific aspects of the alcohol policy across sectors, including road transport and safety, liquor licensing, and treatment and rehabilitation services (Table 1). We did not have any respondent decline to participate, but where one was unavailable, they referred us to another person to interview.

### Data collection

We collected data through 25 face-to-face semi-structured key informant interviews (KIIs) targeting policymakers and implementers. Our total number of KII was guided by the principle of data saturation, whereby we stopped any further interviews once there no new perspectives. The research team developed the interview guide based on the review of policy analysis and implementation literature (Appendix 1). Interview questions were grouped around thematic areas of policy content, process, and factors shaping implementation. Each interview lasted from 30 to 45 min, was recorded in English and transcribed verbatim. Only one participant declined to be recorded, so we took notes during that interview.

### Data analysis

We used both inductive and deductive manifest thematic analysis [22]. In the initial deductive phase, we grouped the main themes according to the six categories of implementation determinants in Bullock’s framework, as explained earlier [18]. The second stage, which was inductive, involved all the authors reading a sample of six transcripts to identify subthemes/ factors that enabled or hindered implementation of the alcohol policy. An initial set of subthemes were developed after a preliminary reading of the transcripts and the field notes. After several discussions within the research team, we then assigned each of the subthemes into their respective determinants of implementation. This process culminated in the development of a coding structure/tree comprising – main and subthemes – which was imported into NVivo 12 Pro Software for the coding of all the transcripts (Table 2). The primary author was responsible for coding and constantly discussed with the team regarding any modifications and updates to the coding structure. After the coding was done, the first author developed the draft coding reports, and each team member was responsible for reviewing and ensuring all themes were accurately captured.

**Table 2:** Implementation determinants of the National alcohol policy in Zambia

Implementation determinants	Emerging themes
Characteristics of the evidence-informed policy	• Comprehensive framework for addressing alcohol-related harms • Unclear policy directives
Networks/Interorganizational relationships	• Restructured coordination committee • Weak collaboration between council and state police
Implementing agency response	• Lack of financial and human resources • Absence of rehabilitation and alcohol treatment services • Misapplication of licences to sell alcohol
Attributes or responses of those affected by the policy	• Community inaction and resistance to alcohol control laws
External environment	• Alcohol framed as an economic development issue • Harmful alcohol use is culturally accepted • Change of governments affected policy ownership • Absence of laws governing illicit alcohol production and unregulated sale of highly potent spirits

### Ethical considerations

This study was part of the PhD project that received ethical approval from the Excellence in Research Ethics and Science Converge Ethics Committee as well as the Zambia National Health Research Authority (ref. no. 2019-Dec-007) in 2021. This approval was renewed in the 2023 (ref. no. 2023 – Jul – 045). Additionally, we obtained permission from the respective ministry permanent secretaries, CSO heads, and government agency managers to conduct the interviews. We provided the participants with information clarifying the benefits and risks of this study, and written informed consent was provided before the interviews. In addition, verbal consent was also provided before the recordings. All interviews were held at the participant’s places of work so there was no requirement for transport refund. To maintain confidentiality, we used participant codes.

## Results

The results are organized according to the study framework’s main thematic areas of policy implementation determinants from Bullock’s framework, and their respective subthemes. These include the characteristics of the evidence-informed policy, network/interorganizational relationships, implementing agency response, attributes or responses of those affected by the policy and the external environment.

### Characteristics of the evidence-informed policy

In this determinant, we highlight stakeholder views regarding how features of the alcohol policy affected its implementation. We identified two subthemes. In the first, the stakeholders perceived the policy as a comprehensive framework for addressing alcohol-related harms. In the second, the stakeholders raised concerns regarding unclear directives in the alcohol policy.

#### Comprehensive framework for addressing alcohol-related harms

Several stakeholders were of the view that the policy provided an overarching framework that supported advocacy and action against alcohol. They explained that it embraced a multisectoral approach, which entailed every sector having a role in its implementation. An official from the MoH shared her perspective.

I think it’s an overarching policy. It provides a framework in which partners or institutions advocate, communicate the effects of alcohol, provide leadership, control illicit alcohol on the market, protect the rights of children and young people, and promote alcohol-related research and development. It also provides for treatment, care, and support. [RMoH004]

#### Unclear policy directives

However, some health and civil society stakeholders felt that alcohol policy statements or measures were broad and diverse. They did not clearly guide decision-making and actions for respective implementers, as one of them expressed:

The policy we are talking about is made of statements. These statements are not clear on what ought to be done. For instance, by Minister of Finance, what exactly are they supposed to do? The statements in the document do not guide. The policy should guide that, for instance, if we’re going to establish rehabilitation centres, what is the specific role of a ministry in implementing this? [RCH007]

### Networks/Interorganizational relationships

Under this determinant, we describe how the nature of relationships and interactions among stakeholder organizations shaped the implementation of the alcohol policy. We identified two subthemes including the restructuring of the policy implementation coordination committee, and the weak collaboration between local councils and state police in enforcing liquor laws.

#### Restructured coordination committee to a lean core group of members

As previously mentioned, the MoH initially oversaw the coordination committee for the implementation of the alcohol policy. However, beginning in 2023, this committee underwent reorganization into a core group of members under new sectoral leadership. According to the stakeholders, the trimming of the committee renewed efforts to implement the policy as it entailed quicker decision-making and implementation of activities. Further, this restructuring gave the responsibility to oversee implementation of the policy to the MLGRD, the current chair of the committee, while the MoH took on the role of secretariat. The stakeholders thought that the MLGRD was most suitable to superintend over the policy because it is the custodian of the principal law that regulates harmful alcohol use – the Liquor Licencing Act. Furthermore, the MLGRD has the requisite structures including the local councils that are responsible for overseeing the awarding of licenses and inspecting of liquor trading facilities.

The Ministry of Health took the upper role in ensuring the development of the alcohol policy. However, we realized that [the] Ministry of Local Government and Rural Development is the one that is mandated to implement the alcohol policy. Also, we were unable to make progress because of too many members in the coordination committee, so we had to reduce it. [RMH009]

#### Weak collaboration between council and state police

According to the local government and civil society representatives, one hurdle to implement the alcohol policy, and particularly the Liquor Licensing Act, stemmed from the lack of understanding and collaboration between the council police and the state police. While their mandate requires cooperating in law enforcement, the state police exhibited a reluctance to address alcohol control matters and preferred to defer such matters to the council police. However, the council police generally lacked authority to enforce alcohol control measures. This situation negatively impacted the implementation of the policy because instances of liquor law violations may occur near a state police station without any corresponding enforcement action.

I should speak about what I can call fragmentation in terms of the mandate holders. For example, the state police and the council people are supposed to work as a team in enforcing the law. However, there are times where the state police feel this a council police matter. [RLCD0012]

### Implementing agency response

This category of determinants highlights some characteristics of the implementing agencies and the behaviours of their frontline staff, respectively. The subthemes include the shortage of financial and human resources, lack of healthcare services for patients with alcohol use disorders, and the misapplication of liquor trading licences in local councils.

#### Limited finances and human resources in implementing agencies

All stakeholders identified the lack of financing for alcohol control activities in the national and sector budgets as a key factor affecting implementation of the policy. This deficiency significantly affected the programming of these activities, particularly at the local government level. Specifically, stakeholders cited challenges such as the inability to purchase fuel to transport inspectors to communities when enforcing the Liquor Licensing Act.

There is no financial commitment pledged for this policy. We must look for money all the time. You can’t implement a policy without funding. That’s a barrier to implementation. Financial resources are required to buy supplies for operatives, such as motor vehicles for patrolling. Enforcement requires liquidity. [RLGD0013]

Lack of human resources was another critical factor affecting implementation of the policy across agencies. The local council and the road traffic representatives reported that they had few inspectors and police officers to cover a large city. This scarcity of staff was exacerbated in rural areas, as stated by a civil society stakeholder.

The ministries that monitor the Liquor Licensing Act have inadequate manpower. Just at a meeting last week, we’re being told the number of council police officers is a drop in the ocean. They’re unable because the magnitude of the alcohol problem requires many human resources for the implementation to be effective. [RCS003]

#### Inadequate rehabilitation and alcohol use disorder treatment services

The MoH and CSO stakeholders noted the lack of treatment and rehabilitation services for people with chronic alcohol use disorders in primary healthcare centres. They pointed out that, despite the presence of a tertiary referral mental hospital in Lusaka, most people avoided it due to the perception that it catered exclusively for mentally ill patients. Further, they deemed the private facilities that provide rehabilitation services expensive and thus inaccessible to the public.

The only sole facility which is affordable is Chainama, but because of the stigma most people shun it until they’re forcibly taken there. This is because it’s viewed as a mental hospital. Once you stabilize, with no alcohol in your systems, you get discharged after a few counselling sessions. [RCS003]

The civil society stakeholders emphasized that women, despite experiencing alcohol use disorders, faced a shortage of rehabilitation services tailored to their needs.

Especially if I may mention for a girl child, even for most of the facilities that are fee paying there is none for ladies. But right now, it’s a struggle, you must fight. There is only one doctor who is trying something. You feel like you out there on your own. It’s a heavy burden if you must deal with a girl suffering from alcohol use issues. [RCS003]

Additionally, stakeholders from the MoH observed that there was a deficiency of trained personnel to provide treatment services to individuals with alcohol use disorders in most health facilities.

At a primary healthcare level, there is nothing. We deliberately tried during the last recruitment to have mental health personnel in each facility, but nothing happened. The people at the district level should understand that alcohol related issues are serious because people die. Facilities need staff that can treat alcohol use disorders. [RMH009]

#### Misapplication of licences to sell alcohol

The stakeholders raised concerns regarding the persistent abrogation of the Liquor Licensing Act in the issuance of liquor trading licences. They stated that, despite the specified requirements of the Act, some alcohol trading businesses that failed to meet the standards were often awarded licences. For example, the granting of licences to sale liquor to some bottle stores enabled them to operate beyond 10:00–19:00 hrs, the prescribed time limit in the Act. Furthermore, there was criticism that the Act appeared to be more focused on charging for registration rather than effectively regulating the distribution and sale of alcohol. An official from the Lusaka City Council explained:

We have a challenge in a situation where a facility by design is supposed to be a bar, but it’s given a restaurant liquor licence, meaning it can play music throughout the night and people will be drinking till the next day. This is one of the challenges that Lusaka is suffering from. The licences were misappropriated, no licences were given rightfully. [RLCC0010]

Representatives of the local authority argued that the huge number of applications of businesses seeking to engage in alcohol trade overwhelmed the licensing committee, which compounded the misapplication of licences. Further, they contended that it was difficult for the licensing committee, which sits intermittently and comprises few staff, to verify huge volumes of applications, which sometimes leads to human errors, resulting in the incorrect awarding of licences.

The number of applicants is overwhelming. I think for Zambia; alcohol consumption has become such a good business. The consumption is high, and many people trade in it. The council committee which issues the licences only meet once in three months in a formal manner to look at the applications. [RLCC0010]

In addition, the awarding of liquor licences was affected by conflicts of interest among the members of the liquor licensing committee. According to the stakeholders, the committee comprised civic and political leaders who also owned liquor businesses, which meant they could not effectively enforce liquor trade measures.

There is a lacuna because the law has not restrained the committee members, who issue the alcohol trading licences, from participating in this same business. Conflict of interest is a major issue – how do they punish themselves if they don’t obey the law? RLCC0010.

### Attributes and responses of those affected by the alcohol policy

Under this determinant, we highlight the behaviours of communities whom the implementation of the alcohol policy impacts, specifically identifying one subtheme that describes their conduct towards the enforcement of the Liquor Licensing Act.

#### Community inaction and resistance to alcohol control laws

The local authority stakeholders conveyed that community members typically refrained from reporting violations of the Liquor Licensing Act, such as the sale of alcohol to underage individuals or establishments operating beyond designated hours. Moreover, they noted that implementing the Act’s provisions posed challenges, as certain community members resisted initiatives undertaken by local authorities. In instances where local authorities closed establishments for breaking the law, the community sympathized with the owners of the establishments. Similarly, stakeholders from the road safety sector encountered occasional resistance from the community while enforcing laws related to drunk driving. A local government representative stated:

The residents are part of the enforcement process. Unfortunately, people always wait for the local authorities. Even if they see something wrong, they will not report to trigger the enforcement process. They complain that the council is not working. Meanwhile, we are not there. Our jurisdictions are huge. [RLCD0012]

Another representative from the road transport and safety agency said:

Any form of enforcement, in many cases, receives backlash, and this is even more the case regarding the perception that the public has of enforcement officers, not only for the road transport and safety agency, but even for other institutions. For example, Zambia Police and the local council. There’s that resistance. [RRTS0017]

### External environment or policy context

This determinant includes factors within the political and soci0-economic environment that may influence implementation of the alcohol policy. The subthemes included the framing of the alcohol problem as an economic issue, excessive drinking being culturally acceptable, the change of government, and an unregulated informal alcohol market.

#### Framing of the alcohol problem as an economic development issue (lucrative business, and source of family income and government revenue)

All stakeholders shared the view that the government’s portrayal of alcohol as an economic issue negatively impacted the implementation of the alcohol policy. They pointed out that the government regarded the alcohol industry as a revenue generator and source of employment. Consequently, authorities exercised caution when enforcing or introducing new regulatory measures including taxation. Further, this perception had led to a widespread proliferation of informal alcohol markets within the Zambian economy whereby people perceived alcohol as a lucrative business that they can easily engage in.

Many young people are unemployed; they have taken to selling of alcohol. For them, it’s a form of employment and they are generating revenue. Some families are entirely reliant on the sale of alcohol, to support themselves, pay rentals, and send their siblings to school. All these people who are selling alcohol are the voters of the government. [RCS002]

#### Harmful alcohol use is culturally acceptable

The stakeholders emphasized that a significant challenge that affected the implementation of alcohol policy was the cultural acceptance of drinking in Zambia. There is a perception of drinking as representing masculinity, generosity, and wealth, and individuals who consume more are celebrated. Further, during traditional and social events, community members encouraged excessive drinking. Although restricted by the policy, because alcohol is deeply entrenched as a social practice, communities did not effectively regulate the age of drinkers within the communities.

It’s cultural because when we drink, I would rather be buying if I have money. At kitchen or wedding parties every weekend there is alcohol. In the homes people stock beers, but that teaches children how to drink. At a funeral there is alcohol too. Any event which does not have alcohol will be poorly attended. [RCH007]

#### Change of government may have affected policy ownership

The stakeholders emphasized that the change of government/political party in 2021 negatively affected the ownership of the alcohol policy, as it was the outgoing government that had developed it. According to their perspective, these circumstances may account for the current government’s sluggishness in implementing the policy. Furthermore, they were concerned that a policy formulated in another administration may not fully align with the aspirations and priorities of the new government.

How is [current Minister of Health] going to disseminate a policy which has a picture of [the former Minister]? When the picture inside is supposed to be that of [the current Minister] because she’s the one who’s in charge now. [RCS0015]

In addition, the stakeholders explained that a new government tends to change the government policy landscape to fit its priorities.

There is now movement towards taking all decision-making to the district so that central government plays a supervisory role. Each district, I think, will do their own thing, employ their own people. So even in terms of treatment services, they are likely to go to back to the council. I think this new dispensation, disturbed the implementation planned in 2018. [RCH007]

#### Absence of laws governing illicit alcohol production and unregulated sale of highly potent spirits

All stakeholders emphasized that the lack of laws governing the illicit alcohol production, and the unregulated sale of highly potent spirits posed a significant obstacle to the implementation of the alcohol policy, particularly in rural and peri-urban areas. Illicit brewing was predominantly carried out in poor households that frequently failed to adhere to essential health standards. Consequently, this rendered the illicit alcohol more affordable and easily accessible to individuals in such settings, including underage individuals. Moreover, the existence of informal markets facilitated the widespread availability of highly potent spirits, often packaged in smaller units like sachets, and sold in virtually every location.

You can’t have a situation where there’s a school and 100 about metres away someone is brewing Kachasu [illicit brew]. They’re selling highly potent alcohol. What do you expect from those guys at school? Immediately they knock off they buy a bottle and put it in the bag. Sometimes they even buy when going to school. [RCS0015]

## Discussion

This study sought to explore factors that facilitate or hinder implementation of the national alcohol policy. We found that this policy was generally viewed by key stakeholders as a comprehensive framework for alcohol control, yet many of its policy directives seemed unclear for the frontline implementers. Further, the restructured alcohol policy implementation coordination committee was said to have enhanced capacity for joint action, however; weak collaboration between key sectors hindered enforcement of alcohol control laws. Moreover, implementing agencies faced several obstacles including insufficient financial and human resources, lack of rehabilitation services, and misapplication of alcohol trading licenses. Community reluctance to support alcohol control laws also affected implementation. Additionally, the framing of alcohol as an economic issue contributed to a culture that was tolerant of harmful consumption, which complicated enforcement of the law. Changes in the government affected policy ownership, while illicit alcohol production and the unregulated sale of traditional and imported spirits further impeded implementation.

### Characteristics of the evidence-informed policy

The recognition of the alcohol policy as a comprehensive framework for the control of harmful alcohol consumption by all the stakeholders provides a supportive environment for policy implementation [23]. However, the ambiguity in some of the policy statements left the implementing agents unclear about their roles and responsibilities. This lack of clarity may be the reason why these agencies adopt minimal to nonrestrictive measures regarding alcohol control [8]. It is also possible that the involvement of the alcohol industry – through sponsoring the policy meetings – during the development of the alcohol policy could have contributed this policy ambiguity. This is because it would be difficult for the government apply restrictive measures to the industry if it is present during the policy process [24]. Indeed, strategic ambiguity, has often been used by the alcohol industry to maintain vagueness in country policies to avoid a restrictive business environment, which may be the case here [25]. For this reason, the WHO warns that countries must be careful to protect the public health interests of citizens from the commercial interests of the alcohol industry.

### Networks/interorganisational relationships

The participants viewed the streamlining of the policy coordination committee and assigning the stewardship to the ministry of local government as a positive development that has potential to improve decision making, communication as well as enhance the organisational capacity to collectively implement alcohol control activities. Indeed, for collaboration to flourish, it is important to get the procedural, institutional arrangements and leadership in place to be able to drive and sustains collective action. According to the participants, the local government ministry poses the infrastructure including the local councils and being the sole enforcers of the liquor Licensing Act puts them in a better position to lead the committee and oversee implementation of the alcohol policy. However, the weak collaboration among government organs like the state and council police contribute to inconsistent policy implementation. Similar findings have been reported in other countries like Australia where the different police authorities were said to be unclear about their responsibilities, which is likely to be the case in Zambia. Perhaps clarifying the responsibilities and accountabilities of key actors in the implementation of the alcohol policy is required.

### Implementing agency response

A significant challenge to the alcohol policy implementing agencies was the lack of financial and human resources, leading to low prioritization of alcohol control activities amid several competing needs [26]. Inadequate budget allocation to alcohol control is a common global phenomenon, with the WHO reporting that less that 2% of global health funds are dedicated to alcohol control despite its negative consequences [1], suggesting a lack of political commitment. This is also evident in the lack of prioritization of the provision of treatment and rehabilitation services for alcohol use disorders in most of the local health facilities, as reported elsewhere [27–31]. Another challenge we noted with the policy implementing agencies was regarding the provision of alcohol-selling licences by the local government to institutions that do not meet set standards, which largely contributes to the illegal and unregulated sale of Alcohol. Many people engaged in the alcohol trading business who have formal authorization are not held accountable, leading to unrestricted access to alcohol, particularly for the young people. This is further compounded by unrestricted alcohol advertising and marketing, which appeals to young people and entices them to consume alcohol, often leading to binge drinking [32].

### Attributes or responses of those affected by the policy

Community reluctance to support enforcement of the Liquor Licensing Act significantly impeded the implementation of the alcohol policy. This behaviour not only weakens the legitimacy and authority of this law but also makes it challenging for authorities to galvanize public cooperation during enforcement. In Canada, the reluctance to support alcohol laws stemmed from a lack of public and retailer awareness of their existence [33]. However, in another study, the lack of clarity regarding the roles of several policy implementing agencies contributed to low public support [26]. Our findings imply a need for proactive engagement, dialogue, and community outreach strategies to address underlying concerns in alcohol laws to enhance policy implementation.

### The external environment

The framing of alcohol as an economic development issue by government negatively impacted policy implementation. The consequence of this framing is noted in the contradicting economic policies that incentivize growth of the alcohol industry over public health [34, 35]. It also contributes to the proliferation of unregulated informal alcohol markets, characterized by the sale of unsafe, cheap, unlicensed and counterfeit alcohol [36]. Although Zambia’s alcohol per capita consumption is relatively low, the prevalence of alcohol use disorders remains high [15]; binge drinking is a huge problem especially among men [16], largely aided by informal markets that sale illicit alcohol. These informal markets erode all forms of accountability, making it impossible to enforce regulatory measures concerning age restrictions, labelling, and quality of alcohol [37]. Additionally, this framing may be contributing to the cultural acceptance of harmful alcohol use by propagating tolerant attitudes and behaviors towards excessive drinking. Our previous study has highlighted that excessive drinking is most common among men [16].

The change of government may have negatively impacted the alcohol policy ownership and its implementation due to a shift in government priorities. The newly elected government of 2021 may have sought to realign policy to its development framework by distancing itself from the previous administration’s policies, which may affect commitment to the alcohol policy. Studies have shown that changes in governments can affect institutional continuity [38, 39], and this may possibly explain the slow policy implementation some years post-enactment. Conversely, a change of government can provide a window of opportunity to align a policy to current political and socio-economic landscape [40]. Indeed, the effect of government change on policy implementation largely depends on the extent to which a new government embraces or diverges from its predecessors.

### Strength and limitations

The use of a qualitative case study allowed us to uncover context-rich information on the factors shaping the implementation of the alcohol policy. The gathering of perspectives from different policy stakeholders not only enriched the interviews but also allowed for a variety of opinions on key themes. During data analysis, the research team’s iterative discussions during data coding enhanced the trustworthiness of our findings. However, the 5- year implementation timeline may have affected the key informants’ recollection of the policy process, introducing some recall bias. Further, the broad nature of policy entails that it may not be clearly bounded, making tracing implementation a challenge, although the use of the study framework helped us to focus on specific determinants. Despite these limitations,t lessons on the implementation determinants of alcohol policies in similar settings.

## Conclusion

The Zambia national alcohol policy faces several implementation bottlenecks including the inability to restrict alcohol marketing and informal markets, to build community action, enforce alcohol laws, allocate resources, or coordinate stakeholders. To enhance the implementation of this policy, the government must build on the stakeholder recognition of the policy framework, and the revamped policy coordination committee under the MLGRD. This should involve empowering the local government to enforce measures that curtail unregulated availability and access to alcohol in local communities through strengthening the liquor licence issuance mechanism. Furthermore, the government should allocate adequate financial and human resources to support alcohol control activities across sectors.

The Zambian experience underscores the complexity of implementing contentious health policies that supposedly have economic benefits and health consequences. Navigating the bottlenecks to implementing such health policies requires a comprehensive approach that addresses regulatory gaps, strengthens community engagement, and enhances collaboration among stakeholders. Additionally, to effectively implement similar health policies not only requires regulatory measures but also robust enforcement mechanisms and sustained political commitment.

## Supplementary Information

Below is the link to the electronic supplementary material.

Supplementary Material 1

Supplementary Material 2

## Data Availability

The raw data generated and/or analyzed during the current study are not publicly available but are available from the corresponding author on reasonable request.

## Abbreviations

CSO: Civil Society Organization
IDI: In-depth interviews
MoH: Ministry of Health
MLGRD: Ministry of Local Government and Rural Development
SSA: Sub-Saharan Africa
WHO: World Health Organization
