# Supplementary material for: Inertia or unanticipated bottlenecks? Exploring stakeholder perspectives on the implementation determinants of the national alcohol policy five years post-enactment in Zambia
Source: Arch Public Health. 2025 Oct 10;83:240. doi: 10.1186/s13690-025-01737-7 (PMC12512930; doi:10.1186/s13690-025-01737-7)
Supplement: Supplementary file 1 — Supplementary Material 1 [file 13690_2025_1737_MOESM1_ESM.docx]

Appendix 1: Interview guide

**Target group**

1. Ministry of Health
2. Ministry of Local Government and Rural Development
3. Ministry of Commerce and Trade
4. Ministry of Finance
5. The civil society organisations

**Purpose of the research**

Thank you for agreeing to do this interview. My name is Adam Silumbwe, the principal investigator of this study. This study aims to identify barriers and facilitators to implementation of alcohol policy in Zambia. To help do that, we would like you to share your experiences and views regarding the current efforts to implement the alcohol policy across sectors.

**Discussion ground rules**

After the completion of the consent process, we will commence the interview. I will read out questions and you are free to skip those you may not be able to respond to. The interview will last an hour. Please note that we shall be recording the information for our analysis.

Have you any questions prior to the interview?

[Turn on the recorders]

I am the interviewer…………………...……………………………. interviewing KII ……………….……Date………….……. Start time……………………End time

**Background Information (**kindly fill in the information below**)**

Sex: Male……………………………………………. Female…………………………………

Occupation and Sector………………………………………………………………………………………………………………………

Experience/years in executing current duty………………………………………………………………………………………

Age at last birthday…………………………………………………………………………………………………………………………….

| **Main question** | **Probe** |
| --- | --- |
| Please tell me about your role regarding alcohol control in Zambia. | - Can you describe to me your experience with the alcohol policy implementation? - In which stage of implementation would you say the policy is? |
| I would like to get you views with regards to the alcohol policy implementation | - What do you think about the content of the alcohol policy? - How feasible and realistic are some of the aspects of the alcohol policy to implement in the Zambian context?   - Which specific aspects of the content do you think are good?   - Which should have been included? - What is your organization doing or have done regarding alcohol policy implementation?   - How is your organization working to implement the policy? - What do you think are the main facilitators to the implementation of the alcohol policy in Zambia?   - Probe for prevailing situations/contexts that make it possible to implement the alcohol policy     - How do you think the policy and legal environment has or is affecting the implementation of the alcohol policy?     - What have been some of socioeconomic and the political factors that facilitate the implementation of the alcohol policy?   - Who have been or are the main actors involved in promoting the alcohol policy implementation?   What role do they play in the implementation of the policy?   - Which do you think are or have been the main barriers to implementation of the alcohol policy in Zambia?   - Probe for prevailing situations/contexts that impede implementation of the alcohol policy     - How would you describe the policy and legal environment and how it affects implementation of the alcohol policy?     - What were some of socioeconomic and the political factors that inhibit the implementation of the alcohol policy?   - Who are the main actors inhibiting the alcohol policy implementation?     - What factors hinder actors’ participation in the alcohol policy implementation? - How do you think we can address these barriers to implementation of the alcohol policy?   - What should be done/what is missing from your perspective? By whom should it be done? - From your own perspective, what do you think has been achieved with regards to implementation of the alcohol policy? |
